# Functional diffusion tensor imaging at 3 Tesla

**DOI:** 10.3389/fnhum.2013.00817

**Published:** 2013-12-03

**Authors:** René C. W. Mandl, Hugo G. Schnack, Marcel P. Zwiers, René S. Kahn, Hilleke E. Hulshoff Pol

**Affiliations:** ^1^Department of Psychiatry, Brain Center Rudolf Magnus, University Medical Center UtrechtUtrecht, Netherlands; ^2^Radboud University Nijmegen, Donders Institute for Brain, Cognition and Behaviour Centre for Cognitive NeuroimagingNijmegen, Netherlands

**Keywords:** white matter, DTI, activation, MRI imaging, task performance and analysis

## Abstract

In a previous study we reported on a non-invasive functional diffusion tensor imaging (fDTI) method to measure neuronal signals directly from subtle changes in fractional anisotropy along white matter tracts. We hypothesized that these fractional anisotropy changes relate to morphological changes of glial cells induced by axonal activity. In the present study we set out to replicate the results of the previous study with an improved fDTI scan acquisition scheme. A group of twelve healthy human participants were scanned on a 3 Tesla MRI scanner. Activation was revealed in the contralateral thalamo-cortical tract and optic radiations during tactile and visual stimulation, respectively. Mean percent signal change in FA was 3.47% for the tactile task and 3.79% for the visual task, while for the MD the mean percent signal change was only -0.10 and -0.09%. The results support the notion of different response functions for tactile and visual stimuli. With this study we successfully replicated our previous findings using the same types of stimuli but on a different group of healthy participants and at different field-strength. The successful replication of our first fDTI results suggests that the non-invasive fDTI method is robust enough to study the functional neural networks in the human brain within a practically feasible time period.

## INTRODUCTION

Neurobehavioral functions depend on a dynamic flow of information between different gray matter brain regions that are interconnected via white matter pathways (Catani and Ffytche, 2005; Mesulam, 2005). Imaging techniques such as diffusion tensor imaging (DTI; Le Bihan et al., 1986; Basser et al., 1994) in combination with fiber tracking (Conturo et al., 1999; Jones et al., 1999; Mori and van Zijl, 2002) allow us to non-invasively study the anatomy of these pathways – but not their activity.

**FIGURE 1:**
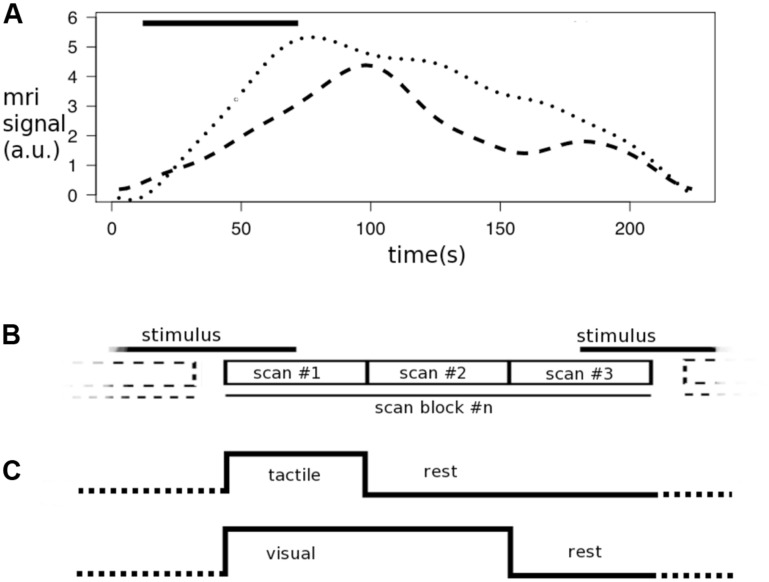
**Response functions and task encoding.** The graphs represent the time course of the measured diffusion weighted MRI signal for a single tactile stimulus (dots) or visual stimulus (dashes; adapted from Mandl et al., 2008) and the scan in combination with a scan block as used in the fDTI experiment. **(A)** Both the tactile stimulus and visual stimulus (bar) started after 12 s with a duration of 60 s. **(B)** The results of the response function experiment show that the maximum of the response function (dotted line) for the tactile stimulus (bar) falls within the first scan of a scan block while the maximum of the response function (dashed line) of the visual stimulus is found between the first and second scan. **(C)** Therefore in the fDTI experiment the tactile task the first scan is contrasted against the second and third scan, while for the visual task the first and second scan are contrasted against the third scan. The signal during the stimulus is constantly increasing (reflecting a reduction in diffusivity in the transverse direction of the tract).

Numerous studies examined the various aspects of using diffusion-weighted MRI in a functional setup (DfMRI) to measure activation in gray matter, using weak (Le Bihan et al., 1986, 1988) or strong diffusion weighting (Boxerman et al., 1995; Prichard et al., 1995; Gulani et al., 1999; Darquie et al., 2001; Jin et al., 2006; Le Bihan et al., 2006; Jin and Kim, 2008; Stroman et al., 2008; Yacoub et al., 2008; Aso et al., 2009; Flint et al., 2009; Kershaw et al., 2009; Autio et al., 2011; Baslow et al., 2012; Branzoli et al., 2013; Tirosh and Nevo, 2013). In our first fDTI study (Mandl et al., 2008) we proposed a non-invasive functional diffusion tensor imaging (fDTI) method that has the potential to detect the white matter fibers that are active during neurobehavioral functioning. In that study, eight healthy participants were scanned on a 1.5 MRI Tesla scanner during a tactile experiment and a visual experiment to assess the validity of the fDTI method. The results of these experiments revealed activation in the contralateral thalamo-cortical tract and optic radiations during tactile and visual stimulation, respectively. Furthermore, these results not only suggested a slowly varying response function for both the tactile and visual stimuli but also that these response functions are different for the different types of stimuli. We speculated that the differences between the response functions could be due differences in perceived intensity of the different stimuli used – the checkerboard stimulus being perceived more intense than the tactile stimulus. **Figure 1A** shows the response functions for a single tactile and a single visual stimulus. In the current study we set out to replicate our previous findings using the same types of stimuli but on a different group of healthy participants with a new fDTI acquisition scheme using a 3 Tesla MRI scanner.

**FIGURE 2:**
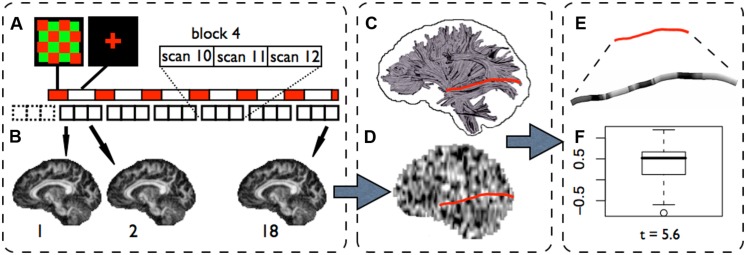
**The fDTI method (visual experiment).** **(A)** In the fDTI experiment 1+6 blocks of 3 DTI scans are acquired. The first block (dashed lines) is a dummy block added to correct for possible scanner onset effects and is disregarded in the further analysis. The necessity for the lag between task and scans is explained in **Figure 2**. For each of the 18 DTI scans an FA map is computed **(B)**. A statistical parameter map (SPM) is computed **(D)** on the FA maps using a general linear model. Fiber tracts are reconstructed for the complete brain **(C)** and, for each tract, the *t*-values found in the SPM along that tract were grouped into a single set of *t*-values **(E)**. For each tract a statistical test (student’s *t*-test) is done **(F)** on the set of *t*-values to test if the average *t*-value found along the tract is significantly (*t* > 5) greater then zero.

Functional diffusion tensor imaging is based on the assumption that task-related changes in fractional anisotropy (Basser and Pierpaoli, 1996; FA) are a sign of local fiber activity. The principle of the fDTI method as applied in this study is outlined in **Figure 2**. In our first fDTI study (Mandl et al., 2008) the conservative non-parametric sign-test formed the statistical basis to test for tract activation. Note that in the present study we used the more familiar parametric t-test because the findings from our first fDTI study suggested that the usage of the *t*-test instead of the sign-test produces very similar results. We hypothesized that morphological changes of glial cells (e.g., oligodendrocytes) induced by activity-related increases in extracellular potassium concentrations could lead to shape changes of the extra-cellular space (ECS; Ransom et al., 1985; Sykova, 2004; Beshay et al., 2005) and, in turn, lead to a measurable increase in FA. Indeed, changes in the diffusion profile due to changes in the ECS in white matter have been shown *in vitro* using diffusion weighted imaging in the rat optic nerve (Anderson et al., 1996). An earlier study (Prichard et al., 1995) reported that electrical stimulation induced significant changes in the diffusion properties of brain tissue in rats. Using intrinsic optical imaging (MacVicar et al., 2002) slowly varying activity-related signal changes were measured in the rat optical nerve, which were attributed to glial cell swelling. However, a study using the real-time tetramethylammonium (TMA^+^) iontophoretic method in combination with intrinsic optical imaging (Sykova et al., 2003) showed that the concentration of TMA+ in the ECS did not change although similar changes in the intrinsic optical imaging signal were measured. Therefore the authors concluded that it was unlikely that glial cell swelling was the primary mechanism for these intrinsic optical signal changes and they suggested that a more plausible explanation may be found in morphological changes of glial cells. Our first fDTI results (Mandl et al., 2008) support this conclusion as the FA-signal changes in the active fibers measured in that study were due to opposite changes in parallel and transverse diffusion coefficients which (for a large part) cancel each other out leading to only small changes in mean diffusivity (MD). If cell swelling was the underlying mechanism for the measured FA-signal changes then an overall reduction in MD is expected because cell swelling would result in a decrease in both transverse and parallel diffusivity in the active conditions. More recent studies (Stroman et al., 2008; Flint et al., 2009) showed that increased levels of potassium lead to changes in both intrinsic optical signal values and MRI proton density measurements for gray and subcortical white matter in rats, suggesting that activity-related changes in the ECS of gray matter as well as white matter can be measured using MRI. In addition, Tirosh and Nevo (2013) showed, using ultra-high field MRI, that neuronal activation results in a 19.5% reduction of the ADC in excised and vital newborn rat spinal cord and concluded that this reversible drop in ADC was due to a reduction in water displacement and could not be related to any hemodynamic effect because the tissue samples were blood-free. Indeed, the contribution of hemodynamic effects to diffusion-weighted functional imaging in gray matter is a topic of extensive research. Various hypercapnia challenge studies show that in gray matter changes in ADC can be measured even when strong diffusion weighting is used. Hypercapnia induces a strong vascular response but no neuronal activity. Because of the strong diffusion weighting, changes in cerebral blood flow and/or cerebral blood volume are not expected to pay a major contribution to the measured change in ADC (Yacoub et al., 2008). However, it was shown that gradient coupling between changes in extravascular susceptibility gradients (i.e., BOLD effect) and the diffusion gradients can result in substantial changes in ADC (Zhong et al., 1991; Hong and Dixon, 1992). These results implicate that DfMRI is not immune to possible hemodynamic effects and posed the question whether or not the reported DfMRI activity-related ADC changes can be fully explained by this gradient coupling effect (Song et al., 1996; Does et al., 1999; Goerke and Moller, 2007; Miller et al., 2007; Lu et al., 2009; Ding et al., 2012; Rudrapatna et al., 2012). Although the results presented in (Stroman et al., 2008; Flint et al., 2009; Tirosh and Nevo, 2013) do provide important evidence that neuronal activation significantly reduces water displacement that can be measured using DfMRI it does not provide information on the relative contributions of the different contrast mechanisms to the measured ADC changes in gray matter. In our first fDTI study we also could not rule out the influence of hemodynamic effects on the measured task-related FA changes in white matter. Indeed, two recent vascular challenge studies (Ding et al., 2012; Rudrapatna et al., 2012) in adult Spraque-Dawley rats using strong diffusion gradients (*b*-values equal or higher than 1000) showed signal changes in white matter ranging between 1 and 2% both in MD and FA. Similar to these results the absolute percent signal change for FA reported in our first fDTI study ranged between 0.98 and 1.45% but in contrast to these results the absolute percent signal change for MD was much lower (between 0.03 and 0.21%) suggesting that the measured ADC changes cannot be readily explained by hemodynamic effects alone and are more in line with possible shape changes of the ECS.

Normal activity-induced ECS changes, however, are expected to be very small as compared to the physiological noise (Gulani et al., 1999) and a reliable detection of the signal change would require a large number of measurements. In the proposed fDTI method we assume that these activity-related glial shape changes extend over the entire active fiber so that a substantial increase in signal-to-noise ratio (SNR) can be achieved by pooling the signal changes over the complete fiber. It is the adoption of a fiber-based statistics -rather than a voxel-based statistics- that enables us to measure the signal within a practically feasible time period.

In our first fDTI study a tactile experiment and a visual experiment were selected for their expected lack of overlap in activated fibers, which allowed us to study both the specificity and sensitivity of the fDTI method. Also the chance of task related motion artifacts was reduced because a subject’s response was not required in either of the tasks. In the present study we used the same types of stimuli but with a new acquisition scheme on a 3 Tesla MRI scanner. For white matter voxels that are part of an *active* fiber, we expected that the FA was higher during the active condition than during the rest condition, thus showing a positive correlation with the task. For the tactile task, activation was expected for the afferent fibers of the thalamo-cortical tracts that connect the thalamus and the contralateral primary sensory area (Kandel et al., 2000). For the visual task, activation was expected mainly for fibers that are bilaterally part of the optic radiation (Kandel et al., 2000).

## MATERIALS AND METHODS

Twelve healthy subjects participated in this study. All experiments presented in this study were approved by the medical ethical committee for human subjects of the University Medical Center Utrecht, the Netherlands, and all subjects signed written informed consent prior to participation. For the tactile stimulus experiment the participants were instructed to keep their eyes closed for the duration of the whole experiment. During the active condition, the palm and fingers of the subject’s right hand were brushed in a random fashion (approximately 1 Hz) by an investigator. In the visual response function experiment the subjects were instructed to look at a red fixation cross that was projected on the center of a screen visible from inside the scanner at all times. During the active condition a red and green checkerboard was shown that alternated at a frequency of 8 Hz.

### IMPROVEMENTS OF THE fDTI ACQUISITION

In the present study we utilized an fDTI acquisition scheme that was improved in several ways to increase specificity by further excluding possible confounding factors (the time settings of this acquisition scheme are detailed in **Figure 1**). (1) We optimized the time settings separately for the tactile stimulus and the visual stimulus because the results of the previous study suggested a considerable time lag in the order of tens of seconds for the visual stimulus, which was not found for the tactile stimulus (**Figure 1A**).

(2) For each type of stimulus, scan slice directions were chosen perpendicular to the expected active tracts in order to minimize the effects of possible motion artifacts that may introduce false positives. If a slice is corrupted because of motion artifacts then for a tract that runs completely through that slice, all points are affected. In contrast, for a tract that runs in the direction perpendicular to that slice only one point is affected. Therefore, the scan slice direction was set in the transverse direction for the tactile stimulus, which is perpendicular to the thalamo-cortical tracts and for the visual stimulus the scan slice direction was set in the coronal direction, which is perpendicular to the optic radiations. (3) The voxel size was set to be anisotropic pointing into the direction of the tracts that are expected to become active in order to reduce partial voluming. (4) In the fDTI experiment 6 blocks of 3 DTI scans were acquired (**Figure 2A**) where stimulus periods (60 s) alternated with resting periods (120 s). Thus, instead of a simple on/off task-design each stimulus period was now followed by two resting periods. In this way possible effects of any periodic signal changes that are not related to the task such as cerebrospinal fluid pulsations (Kao et al., 2008) were minimized.

(5) For both tactile and visual fDTI experiments the stimulus period was shifted with respect to the corresponding acquisition. Now a stimulus period starts at the middle of the last scan of a block and stops at the middle of the first scan of the consecutive block. This shift between the start of the stimulus period and the start of the DTI scan(s) associated with activation was added for two reasons.

First, the time-course experiment described in our first fDTI study showed a slowly varying response function for both the tactile and the visual stimulus (see **Figure 2A**), which was later also reported for gray matter (Baslow et al., 2012). Because of the slowly varying response function the expected signal maximum now falls in the first DTI scan period for tactile stimulus and in the middle of the first and second DTI scans for the visual stimulus. Second, effects of possible faster varying signal changes (such as signal changes due to task-related head motion or blood oxygen-level dependent, BOLD signal) now affect activation and rest DTI scans equally, thereby canceling each other out. Because both activation and rest DTI scans were acquired partially during an activation period (**Figure 1A**).

(6) Rudrapatna et al. (2012) argued that measures derived from the diffusion tensor (e.g., FA) may be devoid of their usual meaning rendering interpretation difficult because the signal is slowly varying during the acquisition of all 7 scans (that is, 1 scan without diffusion weighting and 6 diffusion-weighted scans). To determine if this non-stationarity of the signal would substantially affect the fDTI measurements we circular shifted the diffusion gradient scheme by one for each subsequent epoch. This “round robin” diffusion gradient scheme is detailed in **Table 1**.

**Table 1:** Gradients settings used in the visual and tactile fDTI experiments.

	Scan block #1	Scan block #2	Scan block #3	Scan block #4	Scan block #5	Scan block #6
Gradient #1	G(1, 0, 0)	G(0, 1/2√2, 1/2√2)	G(1/2√2, 0, 1/2√2)	G(1/2√2, 1/2√2, 0)	G(0, 0, 1)	G(0, 1, 0)
Gradient #2	G(0, 1, 0)	G(1, 0, 0)	G(0, 1/2√2, 1/2√2)	G(1/2√2, 0, 1/2√2)	G(1/2√2, 1/2√2, 0)	G(0, 0, 1)
Gradient #3	G(0, 0, 1)	G(0, 1, 0)	G(1, 0, 0)	G(0, 1/2√2, 1/2√2)	G(1/2√2, 0, 1/2√2)	G(1/2√2, 1/2√2, 0)
Gradient #4	G(1/2√2, 1/2√2, 0)	G(0, 0, 1)	G(0, 1, 0)	G(1, 0, 0)	G(0, 1/2√2, 1/2√2)	G(1/2√2, 0, 1/2√2)
Gradient #5	G(1/2√2, 0, 1/2√2)	G(1/2√2, 1/2√2, 0)	G(0, 0, 1)	G(0, 1, 0)	G(1, 0, 0)	G(0, 1/2√2, 1/2√2)
Gradient #6	G(0, 1/2√2, 1/2√2)	G(1/2√2, 0, 1/2√2)	G(1/2√2, 1/2√2, 0)	G(0, 0, 1)	G(0, 1, 0)	G(1, 0, 0)

*G(x, y, z) is the gradient direction vector where x points in the subjects right–left direction, y points in anterior–posterior direction and z points in the inferior–superior direction. For each subsequent scan block the set of six gradients is rotationally shifted by one.*

### SCAN ACQUISITION PARAMETERS

For each experiment a separate T1-weighted scan, two conventional high-resolution DTI scans, and an fDTI scan were acquired. All scans were acquired on a Philips Achieva 3 Tesla whole-body MR scanner (Intera Achieva, Philips, Best, The Netherlands) using an eight-channel head coil.

A sagittal 3D T1-weighted whole brain scan was acquired for anatomical reference, inter-subject registration, the creation of a white matter mask and visualization of the results (acquisition matrix 304 × 299 × 200; FOV = 240 mm × 240 mm × 160 mm; TR = 10 ms; TE = 4.6 ms; flip angle = 8 degrees; SENSE parallel imaging in both phase encoding directions = 1.5; total scan duration 602 s). Next, two conventional transverse Stejskal-Tanner diffusion weighted single shot spin-echo, echo planar imaging (SS-EPI) DTI scans were acquired (FOV = 240 mm × 240 mm; acquisition matrix 128 × 128; reconstruction matrix 128 × 128; slice thickness 2 mm; 75 consecutive slices; flip angle = 90 degrees; TE = 68 ms; TR = 7047 ms; parallel imaging SENSE factor = 3; total scan duration 268 s, no cardiac gating; Mandl et al., 2008; van den Heuvel et al., 2008). The second conventional DTI scan differs from the first one in that the k-space readout direction (anterior–posterior) is reversed. These conventional DTI-scans were used for reconstruction of the fibers (**Figure 2C**). The functional time series of DTI scans (the fDTI set) were acquired during the execution of an alternating sequence of a neurobehavioral task and a resting condition. For the tactile experiment a total of seven sets of three transverse SS-EPI DTI scans (acquisition matrix = 96 × 96; FOV = 240 mm; 30 slices; slice-thickness = 7 mm; no gap; TE = 78 ms; TR = 6000 ms; parallel imaging SENSE factor = 3; 90 degrees flip angle; 6 non-collinear diffusion gradient directions with b-factor = 1000 s/mm^2^ and 2 scans without diffusion gradients; scan duration per DTI scan = 60 s) were collected. No cardiac gating was used as it would lengthen the experiments. For the visual experiment the fDTI scans were acquired in coronal direction with otherwise identical parameter settings. The first set is a dummy set that was added to eliminate possible scanner onset effects (e.g., gradient heating). Per set the order of the diffusion gradient directions was circular shifted (**Table 1**). For the dummy set the same ordering of the diffusion gradients was used as for the first real set. For each set one stimulus period was presented. A stimulus period started at the middle of the acquisition period of the last DTI scan of the previous block and stopped at the middle of the acquisition period of the first scan of the current block (**Figure 2A**). The subjects left the scanner room for at least 15 min to rest between the two experiments. The order of the fDTI experiments (first tactile then visual or vice versa) was balanced and randomized.

### FIBER TRACKING

The two conventional DTI scans were combined to remove susceptibility-induced distortions (Andersson et al., 2003). After correction (Andersson and Skare, 2002) of the gradient-induced distortions and subject motion the diffusion tensors were computed using robust tensor estimation (Chang et al., 2005) based on M-estimators yielding a single DTI volume. The DTI volume was used to reconstruct the fiber tracts for the whole brain with the FACT algorithm (Mori et al., 1999). Parameter settings: minimum FA > 0.15, maximum angle between current major eigenvector and previous major eigenvector <37 degrees, average maximum angle between current major eigenvector and major eigenvectors of neighboring voxels (*R*-value) < 37 degrees, minimum fiber length 50 mm, number of fiber starting points per voxel = 8. The fiber tracking was constrained within the white matter by using a white matter mask that was created on the T1-weighted scan using SPM2 (Wellcome Department of Cognitive Neurology, London, UK) and overlaid on the conventional DTI set. The rigid transformation needed to align the T1-weighted scan with the conventional DTI volume was computed between the T1-weighted image and the diffusion unweighted scan from the (susceptibility corrected) conventional DTI image with the ANIMAL software package (Collins et al., 1995) using mutual information as a similarity metric.

### STATISTICAL ANALYSIS

Because the measured FA values and noise characteristics may vary considerably at different positions along a fiber, the effect size of a task-related signal change is not constant for all voxels that are part of the fiber tract. Therefore statistical tests that assume equal effect sizes for all parts of the fiber are not suited to test for fiber activation. In the fDTI method the comparison between active and rest FA values is performed *per voxel* using a general linear model (GLM). The resulting *t*-value then represents the difference between activation and rest independent of the effect size. The results for all voxels together form a statistical parameter map (SPM) that is used to test for task-related fiber activation (**Figure 2D**). The computation of the SPM is described below. For each of the reconstructed fibers (**Figure 2C**) the set of *t*-values in the SPM that coincides with the fiber is selected (**Figure 2E**) and tested whether its mean *t*-value is significantly greater than zero (here we used a fixed threshold of *t* > 5, uncorrected; **Figure 2F**). Because of the differences in response functions for tactile and visual stimuli, different task encoding regressors for the reconstruction of the SPM were used in the tactile and visual experiment (**Figure 1**). The first DTI scan (activation) was compared with the second and third DTI scan (rest) of the set for the tactile stimulus, while for the visual stimulus the first and second DTI scan (activation) were compared to the third DTI scan (rest).

### STATISTICAL PARAMETER MAP CREATION

To correct for inter-scan subject motion all different diffusion weighted (and unweighted) volumes were rigidly aligned (using cross-correlation as similarity metric) with their counterparts from the first DTI scan. Next, the FA maps were computed for each of the 18 registered DTI scans. For each FA time series (i.e., the FA value of a single voxel followed over time) a *t*-statistic was computed using a GLM with two regressors. The first regressor encoded for activation (activation = 1, rest = 0). The second regressor (with linear increasing values between 0 and 1) was added to correct for effects of possible scanner drift. The results of the first regressor form the SPM that is used to test for activation of the entire fiber tracts (**Figure 2D**). Realignment of the SPM with the reconstructed tracts was done using a linear transformation that was computed between the average diffusion unweighted scan of the conventional DTI scan and the fDTI set using cross-correlation as a similarity metric.

### ACCUMULATED RESULTS

The accumulated results here were created analog to accumulated results presented in (Mandl et al., 2008). In short, for each subject a binary map of the complete set of voxels that coincides with the active fibers found is placed in one common space using the linear transformation that registers the subject’s anatomy scan with the Montreal Neurological Institute MNI-305 template. Each of the transformed sets is then blurred with a 3-dimensional Gaussian kernel with a full width at half maximum of 7 mm followed by a threshold at a value of 0.1 yielding a second binary map. Finally these binary maps of the subjects are accumulated and overlaid on the subjects’ average anatomy. Thus the value of a (colored) voxel represents the minimum number of subjects for which an active fiber can be associated with that voxel.

## RESULTS

**FIGURE 3:**
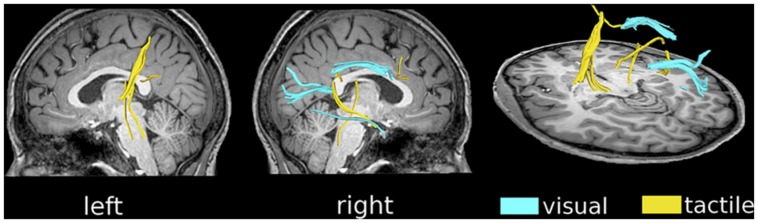
**fDTI results for a single subject.** Tracts that were found active during the visual task (blue) and the tactile task (yellow) using the fDTI method. During the tactile task, activation was found predominantly contra-laterally for the thalamo-cortical tracts running from the thalamus to the primary sensory cortical area. Activation during the visual task was found, amongst others, for tracts that are part of the optic radiation and the genu of the corpus callosum.

**FIGURE 4:**
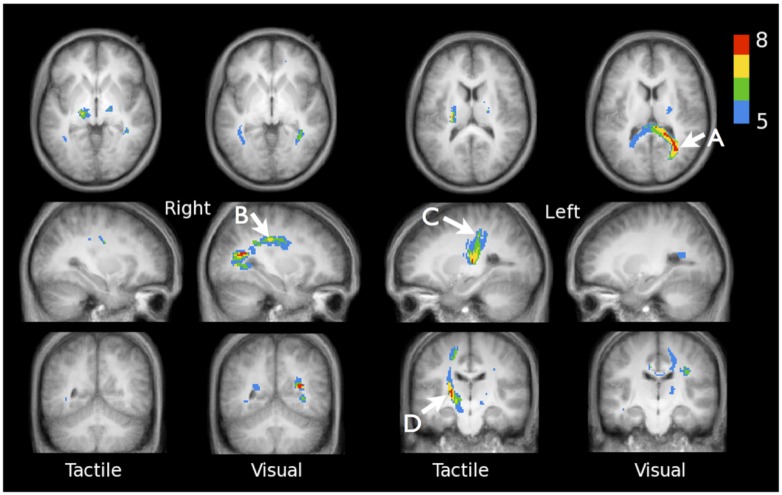
**Accumulated fDTI results of all 12 individuals for the tactile and visual tasks.** For both tasks the cumulative fDTI results were computed and overlaid on the subjects’ average anatomy. The value of a (colored) voxel represents the minimum number of subjects for which active fibers are found at that position. Visual activation is found for tracts that are part of the forceps major **(A)** MNI-coordinate (31, -58, 16), superior longitudinal fasciculus **(B)** MNI-coordinate (31, -24, 34), and at positions that correspond with the optic radiations. The results show that the majority of the tactile activation is found in the contralateral tracts connecting the thalamus and sensory cortex. Maximum cumulative values are found at MNI-coordinates (-27, -30, 34; **C**) and (-19, -22, -3; **D**).

**Figure 3** shows the active fibers found for a single subject in the tactile fDTI experiment and the visual fDTI experiment. For each task, the results of all individuals were placed in a common space to study the cumulative activation patterns (**Figure 4**). For the tactile experiment contralateral activation of the left sensory thalamo-cortical tract was found. A maximum cumulative value 7 of 12 (indicating the number of subjects that had an active fiber running through that voxel) for the superior part of the left thalamo-cortical tract was found at (-27, -30, 34) in MNI space. The maximum cumulative value 8 (of 12) for the inferior part of the left thalamo-cortical tract was found at MNI coordinate (-19, -22, -3).

For the visual experiment bilateral activation was found predominantly in the optic radiations. The maximum cumulative value 8 (of 12) for the visual task was found at MNI coordinate (31, -58, 16), which is located in the forceps major (according to the JHU white matter tractography atlas; Hua et al., 2008). More superior, a local maximum cumulative value 7 (of 12) was found at MNI location (31, -24, 34), in the right superior longitudinal fasciculus.

The mean percent signal change found for the voxels part of the active fibers for the tactile task was 3.47% (SD = 1.86), -0.10% (0.44), -0.65% (2.01), 0.09% (0.57) and 0.10% (0.18) computed for the FA, MD, transverse diffusivity, parallel diffusivity and the diffusion-unweighted signal (B0), respectively. For the visual task the mean percent signal change was 3.79% (1.79) for the FA, -0.09% (0.70) for the MD, -0.35% (1.03) for transverse diffusivity, 0.63% (0.86) for parallel diffusivity and 0.14% (0.25) for the B0 signal.

## DISCUSSION

Here we report activation measured along white matter tracts in the brains of healthy volunteers during tactile and visual stimulation using fDTI on an MRI scanner operating at 3 Tesla. This replication of our previous fDTI results on a different group of healthy participants using a different MRI scanner operating at 3 Tesla can be seen as a further indication that the fDTI method can successfully be applied to measure white matter activation. The white matter activation patterns that we found are very similar to the activation patterns found in our first fDTI study. For the tactile stimulus, task-related changes in FA-values were found in the contralateral sensory thalamo-cortical tract. A maximum cumulative value 7 was found for the superior part of the left thalamo-cortical tract at MNI coordinate (-27, -30, 34) which is in good agreement the results of a recent fMRI study that used a tactile stimulus similar to the one we used (Jang et al., 2013). That study reported that for stimulation applied to the palm of the right hand of 15 healthy volunteers the peak activation value was found at MNI coordinate (-38, -24, 60), which is located in the primary sensory motor cortex.

For the visual stimulus task-related changes in FA-values were predominantly found in the optic radiations and forceps major. The latter contains fibers that connect homotopic visual regions. Interestingly, activation was also found in the right superior longitudinal fasciculus – a major fiber bundle that connects to the intraparietal sulcus (Uddin et al., 2010). The intraparietal sulcus is a structure that has been associated with perceptual motor-coordination, visuo-spatial working memory and visual attention (Swisher et al., 2007). Structural changes in white matter adjacent to this structure that were induced by training of a complex visuo-motor skill have been reported previously (Scholz et al., 2009). In that study no significant correlation was found between these structural FA changes and training progress or performance level, and it was therefore suggested that these FA changes could be more related to the amount of time spend training. Our results suggest that visual attention may also play a role in the reported structural FA changes because in our visual task no learning was involved.

The aim of our first fDTI study was to demonstrate that it was possible to non-invasively measure task-related activation in the brains’ white matter. Based on the results from that study we hypothesized that subtle task-related morphological changes of glial cells resulted in measurable FA changes. However, we could not exclude possible hemodynamic contributions to the measured signal. Indeed, two recent vascular challenge studies (Ding et al., 2012; Rudrapatna et al., 2012) showed measurable changes in the order of 1–2% for the both FA and MD in white matter. Their results showed that the changes for MD where of the same size (or higher) than the changes in FA. Also, changes in the diffusion-unweighted signal (B0) were even more pronounced (this was also reported in humans; Kershaw et al., 2009). In contrast, the results from our first fDTI showed that the mean percent signal change in MD computed for all voxels part of the active fibers was much smaller then the mean percent signal change in FA. The same pattern was found in our current study with the mean percent signal change in FA for the tactile task being 3.47% and for the visual task 3.79% while for the MD the mean percent signal change was only -0.10% for the tactile task and -0.09% for the visual task. In addition, for the B0 signal the corresponding mean percent signal change was 0.10% for the tactile task and 0.14% for the visual task. This pattern of a relatively large mean percent signal change for FA compared with the mean percent signal change in MD and B0 is more in line with the hypothesized activity-related morphological glial cell changes and suggests that the observed signal changes cannot be explained by hemodynamics alone (Song et al., 1996; Does et al., 1999; Goerke and Moller, 2007; Miller et al., 2007; Lu et al., 2009). Thus, a possible hemodynamic contribution (and if present by itself a valid and interesting contrast mechanism for measuring activation in white matter) may not fully explain the measured fDTI signal. Other confounding factors that are not directly related to neuronal activation may also have contributed to the measured changes in FA. For instance, fMRI studies showed that changes in respiration patterns can introduce magnetic susceptibility changes leading to artificial activation patterns found in white matter (Windischberger et al., 2002). In our approach, however, the role of this type of magnetic susceptibility changes is probably quite limited because of the introduction of the temporal shift for the stimulus onset (**Figure 1A**). Because of this shift, this type (and other types) of fast varying task-related signal changes (e.g., BOLD contrast) are largely canceled out. Furthermore, the stimulus design used in this study (1 stimulus period followed by 2 rest periods) reduces the possibility that any type of sinusoid signal fluctuations could interfere with task-related signal changes and therefore contribute to the measured changes in FA-signal.

Diffusion-weighted MR acquisitions are known to be very sensitive to motion artifacts (e.g., cardiac pulsation, voluntary subject motion). In our first fDTI study we used the same 2-dimensional axial DTI acquisition for both the tactile and visual experiment. In a 2-dimensional sequence, motion artifacts typically affect the quality of the scan at a slice level. For tracts running in parallel with the slice direction (such as optic radiations) the sensitivity to motion artifacts is therefore relatively high because large parts of the tract run trough one single slice. This in contrast to tracts running perpendicular to the slice direction (such as the thalamo-cortical tracts) because here each slice only contains a small part (1 or 2 voxels) of the complete tract. In the current experiments we used a coronal slice direction for the visual experiment to eliminate this difference in sensitivity to motion artifacts. The fact that the results of the current study are similar to the results of our first fDTI study suggests that the chosen slice direction is not a dominant factor in the experimental setup.

Despite the application of the temporal lag to maximize the measured signal change and the increased SNR due to the increased main magnetic field strength the bilateral activation predominantly found for the visual experiment in the optic radiations appears to be less pronounced at 3 Tesla than the activation found for the visual experiment in the optic radiations at 1.5 Tesla (Mandl et al., 2008). This difference in sensitivity may be explained by the lower number of stimulus periods (6) in the 3 Tesla experiment as compared to the number of stimulus periods (12) in the 1.5 Tesla experiment. Moreover, due to the smaller voxel size at 3 Tesla (43.75 mm^3^) compared to the voxel size at 1.5 Tesla (64 mm^3^) the SNR between a single fDTI scan 1.5 Tesla is comparable to a single fDTI scan at 3 Tesla because the expected increase in SNR with a factor of √2 due to a doubling of the main magnetic field strength (3 Tesla vs 1.5 Tesla) cancels out. Also, because of the thick slices used in the 3 Tesla experiments (7 mm) internal dephasing may contribute to a reduction of the SNR.

In the current study we applied a circular shift to the gradient direction settings for subsequent fDTI scans to determine if the non-stationarity of the response function during acquisition (Rudrapatna et al., 2012) would substantially alter the results. The successful replication of the results of our first fDTI study suggests that the effect of a non-stationary signal on the detection of white matter activation is limited although we cannot exclude that additional variation introduced by the use of this circular shift lowered the sensitivity of the fDTI method.

Both 1.5 and 3 Tesla experiments were specifically designed to maximize the specificity of the fDTI method to minimize the change of spurious fiber activation because the main purpose of these experiments was to assess the feasibility of the fDTI method. Further experiments are needed to determine the optimal stimulus and MRI parameter scanner settings to optimize the sensitivity of the fDTI method.

In conclusion, we replicated the results of our previous fDTI study using the same types of stimuli but with an improved scan acquisition scheme on a different group of healthy participants using a 3 Tesla MRI scanner. This replication of our previous fDTI results suggests that the fDTI method can be applied within feasible time period and is robust enough to become a valuable tool that can help us to get a better understanding of the dynamics of functional neural networks in the human brain.

## Conflict of Interest Statement

The authors declare that the research was conducted in the absence of any commercial or financial relationships that could be construed as a potential conflict of interest.
